# Application of the Jigsaw Puzzle Flap Based on Freestyle Perforators to Repair Large and Deep Ulcers on the Buttocks

**DOI:** 10.3389/fsurg.2022.739250

**Published:** 2022-04-13

**Authors:** Xiaoming Chen, Biao Huang, Haitao Xiao, Lu An, Wenxing Su, Daojiang Yu

**Affiliations:** ^1^Department of Plastic and Burn Surgery, The Second Affiliated Hospital of Chengdu Medical College, China National Nuclear Corporation 416 Hospital, Chengdu, China; ^2^Department of Burn and Plastic Surgery, West China Hospital, Chengdu, China

**Keywords:** buttocks, decubitus, reconstruction, freestyle perforator, jigsaw puzzle flap

## Abstract

**Background:**

Decubitus ulcers are common skin injuries in plastic and burn surgery departments, usually occur in patients with a long disease course and poor underlying health. Designing a reconstruction procedure with safety blood supply to a large volume soft tissue and resulting in minimal trauma is a priority for surgeons.

**Methods:**

The free-style perforators on the potential donor sites surrounding the ulcers were detected by Doppler, and the area of the ulcer was divided into several sections based on the location of pre-design perforator flaps. According to the insertion point of the perforators, small V-Y advancement flaps, propeller flaps and rotation flaps pedicled with freestyle perforators were formed and moderately modified during surgery. All of the small flaps were transplanted from donor sites to the defect and reassembled into a new composite flap to repair the ulcer. The donor sites were directly closed. The area of the flaps ranged from 7.0 × 10.5 cm to 8.0 × 22.0 cm and the diameter of the pedicle perforators ranged from 0.5 to 4.0 mm.

**Results:**

In 30 patients, 65 flaps were constructed, and all of the flaps survived with direct closure of all donor sites. One case with effusion healed 1 month postoperatively through draining and application of a mild pressure dressing. After a 3–24 months follow-up period, all of the patients were satisfied with post-operative function and appearance, and only one case had a local recurrence 6 months postoperatively.

**Conclusion:**

The jigsaw puzzle flap based on freestyle perforators can repair the large skin and soft tissue defects caused by decubitus ulcers on the buttocks, with direct donor flap area closure. This method is easy to perform with a safe blood supply and minimal trauma resulting from the avoidance of microvascular anastomosis and the conventional myocutaneous flap.

## Introduction

According to the NPIAP, EPUAP, and PPPIA, the prevalence of pressure ulcers (PU) in UK is about 21.8%. In addition, a study on the prevalence of PU in the Netherlands surveyed more than 38,000 patients. The results showed that the prevalence rate of PU was close to 13% in university-affiliated hospitals, 23% in general hospitals, 30% in sanatoriums and 12% in home care. Similar research data can be found in other countries. Such a high prevalence rate has brought a huge financial burden to country (1, 2). With the aging of society and changes in the disease spectrum, incidence of decubitus ulcers is increasing. The prevalence rate of stage III and stage IV pressure ulcers in China is 13 to 14%. These Patients are commonly treated in plastic and burn surgery departments in China (3, 4). Repair of decubitus ulcers with large and deep cavities requires soft tissue filling with a large volume and good blood supply. Local gluteal myocutaneous flaps and distal free myocutaneous flaps are commonly used (5–8). However, patients with decubitus ulcers usually have a long disease course, copious wound exudation, a large loss of protein, and poor underlying health. Therefore, these patients are poor candidates for myocutaneous flap or microanastomotic procedures to treat a large and deep ulcer. The wounds with deep pressure sores and large defects after debridement, the first choice is to use skin flap transplantation to repair. The skin flap not only has the thickness of full-layer skin, but also has subcutaneous tissue. After the repair and healing of the flap, the thickness of the healed wound is the same as that of normal skin, and the skin has good compression and wear resistance (9, 10). When encounter a large and deep defect wound, the recovery time of skin flap transfer is significantly shorter than that of single negative pressure treatment or conservative dressing change (11). For patients with this type of decubitus ulcer, designing a reconstruction procedure with a safe blood supply to a large area resulting in minimal trauma is a priority for plastic and burn surgeons.

With evolving research on the blood supply of perforator flaps and perforator location techniques, a novel perforator flap called the freestyle perforator flap has been rapidly developed to provide a reliable blood supply and less trauma while avoiding vascular anatomical variations (12–17). The key to the success of the freestyle perforator flap is that surgeons performing it forgo consideration of traditional anatomical landmarks and vascular variations during flap design and formation, instead using the detected signal of the perforator from Doppler and the characteristics of the donor sites to freely design flaps matching the defect (18–20). The branches of the perforator, rather than the main blood vessel, must be dissected when separating the vascular pedicle of the freestyle perforator flap. Advantages of this technique include shorter operation time, less trauma, and direct closure of donor sites. Combined with the concept of the “economic flap” proposed by Professor Yixin Zhang (21, 22) and the anatomical characteristics of the large amount of tissue around the defect on the buttocks, designed two or three local freestyle perforator flaps transferring to the defect, which made it possible to re-embed or tile a new flap with a larger area and volume. Therefore, we applied the jigsaw puzzle flap based on the freestyle perforator to repair decubitus ulcers with large and deep cavities on the buttocks. From December 2013 to January 2020, we treated 30 cases and achieved satisfactory results.

## Materials and Methods

### Patient Information and Ethical Approval

Thirty patients (18 males and 12 females) underwent jigsaw puzzle flap procedures based on the approach of the freestyle perforator and the concept of using “perforasomes” to repair decubitus ulcers with large and deep cavities on the buttocks. Patient ages ranged 50–81 years, averaging 65. The sizes of the defects were measured: 6.0 cm × 8.5 cm × 2.5 cm to 10.5 cm × 11.0 cm × 4.0 cm. The minimum flap size was 7.0 cm × 10.5 cm, and the maximum was 8.0 cm × 22.0 cm. There were 41 perforator propeller flaps, 19 rotation flaps, and five V-Y advancement flaps. Adjacent zones with the fastest blood flow velocity (more than 2.5 cm/s) and the best soft tissue mobility were selected as the preferred donor area for freestyle perforator flaps. All of the flaps were mobilized to the defect to create a new, large jigsaw puzzle flap that repaired the defect with no tension. The clinical features of the patients are summarized in Table 1.

**Table 1:** Clinical characteristics of decubitus ulcers patients.

**Characteristics**	**Cohort (*N* = 30)**
N (%)	
Age	
≤60 years	14 (47)
>60 years	16 (53)
Gender	
Male	18 (60)
Female	12 (40)
Site of decubitus ulcers	
Sacrococcyx	19 (63)
Trochanter	6 (20)
Ischial tubercle	5 (17)
Bedridden reason	
Fraility due to geriatric disease	20 (67)
Car accident injury	8 (27)
Fall from a high altitude	2 (6)
Combine with bone exposure	
No	6 (20)
Yes	24 (80)
Average postoperative stay	
≤ 2 weeks	26 (87)
2–3 weeks	3 (10)
>3 weeks	1 (3)
Ulcer recurrence	
No	29 (97)
Yes	1 (3)
Flap type (*N* = 65)	Total flap = 65
Perforator propeller flaps	41 (63)
Rotation flaps	19 (29)
V-Y advancement flaps	5 (8)

Case inclusion criteria: (1) decubitus ulcers caused by traumatic diseases such as paraplegia; (2) caused by dyskinesia and long-term bedridden caused by senile diseases such as Parkinson's disease; (3) long-term bedridden caused by iatrogenic injuries. Exclusion criteria: (1) patients with severe medical diseases (such as diabetes, arteriosclerosis, autoimmune diseases, etc.) or heart, lung, liver and kidney dysfunction, assessed by surgeons to be unable to tolerate surgery; (2) patients who have a long history of smoking and are still smokers and alcoholics; (3) pregnant and lactating women; (4) mental illness, lack of insight, inability to express and collaborate; (5) other reasons that are not suitable for this operation.

This study was approved by the institutional review board and ethics committee of the hospital (the full name of the institution has been removed for submission) and adhered to the tenets of the Declaration of Helsinki. Written informed consent was obtained from each patient and their families. We provide it as supplementary materials.

### Operative Technique

#### Mapping the Location of Freestyle Perforators

The first step in designing a jigsaw puzzle flap was to locate all of the sizable freestyle perforators in areas adjacent to the defect. All of the skin-penetrating positions of freestyle perforators in this area on the buttocks were identified, and the signal amplitude and blood flow velocity were recorded. The perforator with the most prominent Doppler signal was used as the pedicle of the flap. As all the perforator flaps have a safe range of blood supply, with one perforator as the pedicle, the adjacent area of the perforasome can be safely supplied through the linking vessel (18–20). It is necessary to mark all the perforators in a wide range before surgery because the axial phase and safe boundary of the freestyle perforator flap must be determined by detecting the positions of adjacent perforators.

Before operation, all the free perforating vessels in the periphery of the defect were detected by portable ultrasonic Doppler blood flow detector (Smartdop45, Japan Co., Ltd.). For the perforating vessels detected through the skin, the perforating vessels with the strongest signal were used as the pedicle of the flap, and the perforating vessels with weak signals were selected to a backup. Then mark the flow velocity (2.5, 5.0, 10.0cm/s).

#### Design of the Skin Flap

The pedicle, axis, and size of the free perforator flap were determined according to the size of the defect to be repaired, the location and distribution of the freestyle perforators detected, and the range of motion of the soft tissue around the defect. Thus, the flap was pre-designed and marked with Methylene Blue Ink.

**Figure 1:**
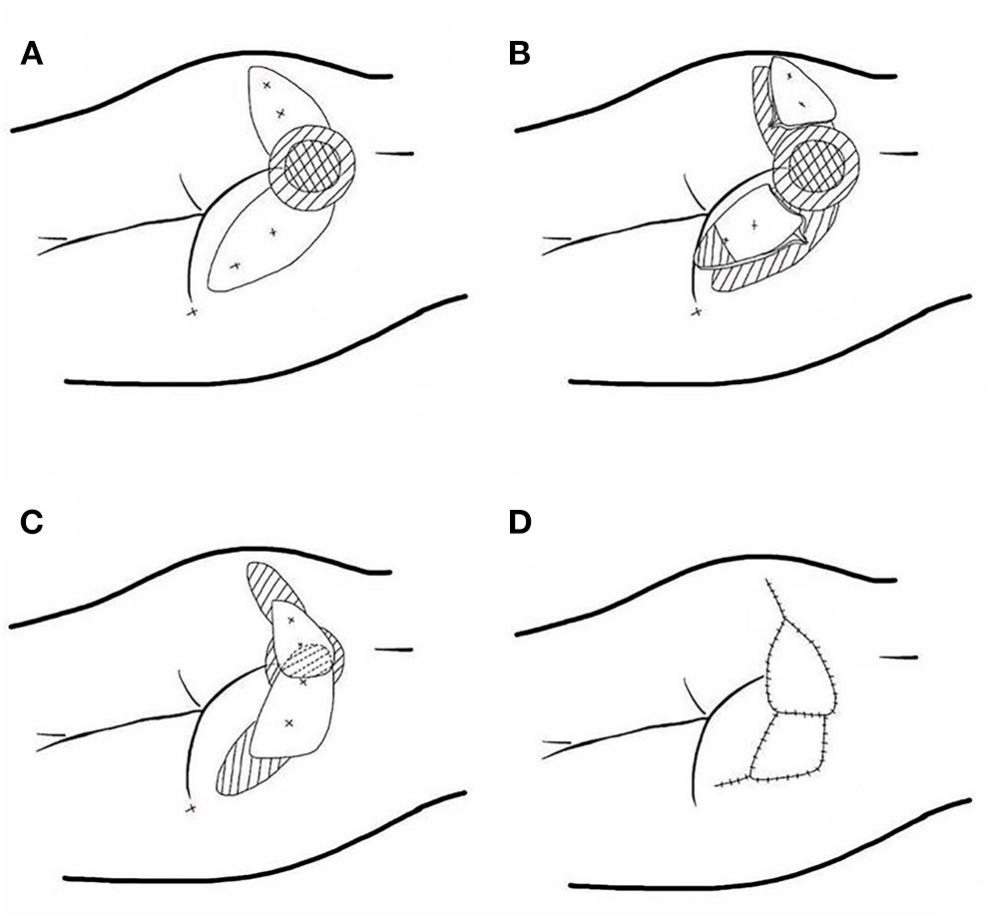
Schematic diagram of the reconstruction of pressure ulcers on the buttocks using a jigsaw puzzle flap based on freestyle perforators. **(A)** The freestyle perforators were detected and the component flaps were designed. **(B)** All of the flaps were sharply dissected and elevated in a subfascial or suprafascial plane and part of the epidermis was removed. **(C)** The flaps were mobilized to the defect to form the three-dimensional puzzle flaps. **(D)** The wound was covered.

**Figure 2:**
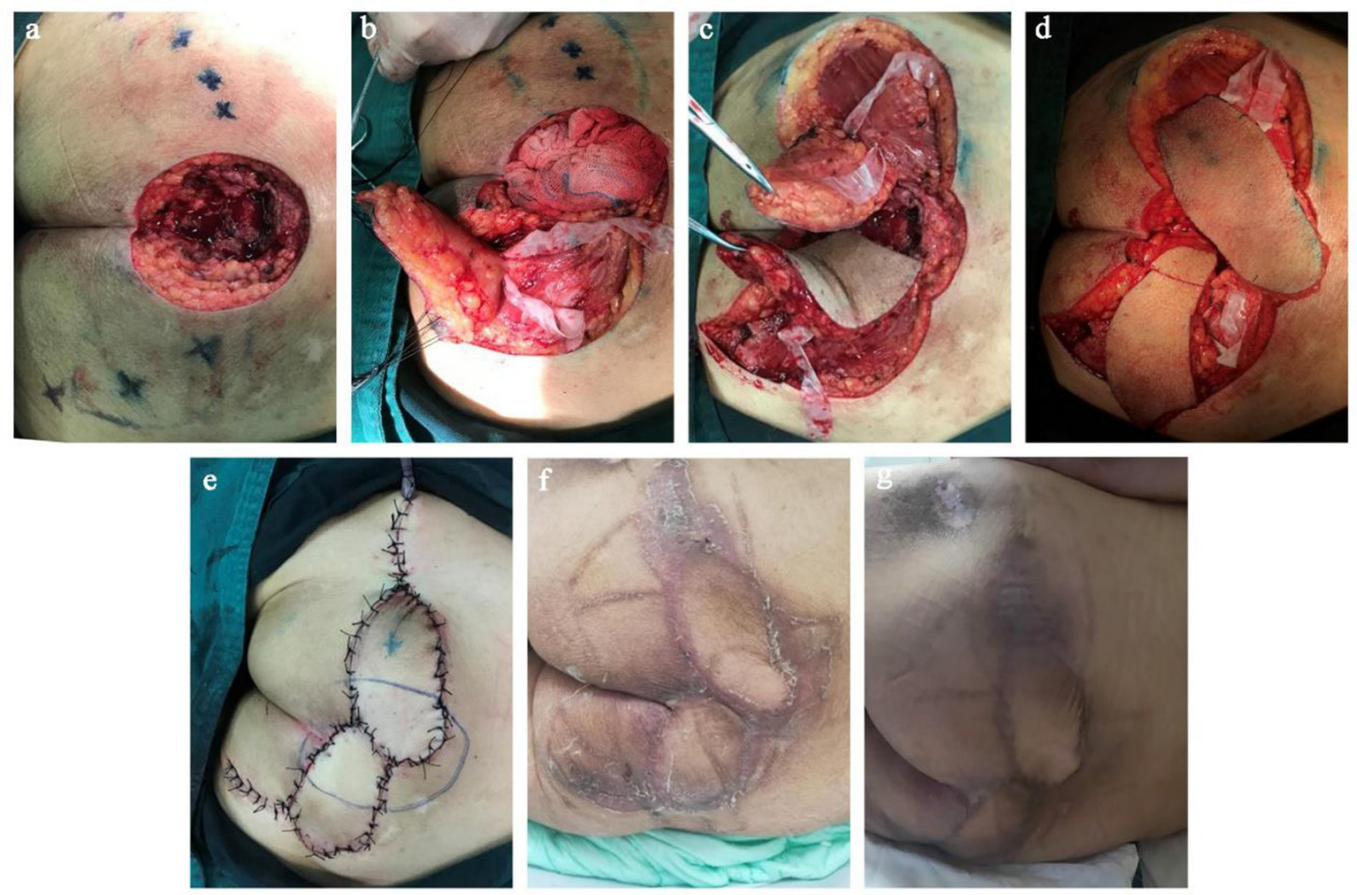
Surgical procedure for repair of decubitus ulcer on the buttocks with a freestyle perforator jigsaw puzzle flap. **(a)** Detection and location the freestyle perforators around the defect using Doppler after radical debridement. **(b)** Dissection of freestyle perforators. **(c)** Component freestyle perforator flaps were harvested. **(d)** Two component flaps were mobilized together and design the epidermis resection range of one flap. **(e)** The new, large jigsaw puzzle flap covered the defect, and the donor sites were closed primarily with no tension. **(f)** The appearance of the surgical site 3 weeks after the operation. **(g)** The appearance of the surgical site 6 months after the operation.

The necrotic and scar tissue around the cystic cavity-type decubitus ulcer were completely removed with meticulous hemostasis. Following the guidelines for constructing a jigsaw flap, the large defect was divided into several small parts, with each component corresponding to a freestyle perforator flap, according to the location of the free perforator detected by Doppler before surgery and the area of the component flap that could be harvested, which was combined with the requirement of direct closure of all the donor sites. We removed part of epidermis of the free perforator flap to form a dermal-fat flap and rotated the flap 180 degrees to fill the deep cavity of the defect. The flap took the location of the skin penetration point (the place where the perforator had the strongest signal) as the rotation point. Then, the dermal-fat flap was lap-jointed with another freestyle perforator flap using “overlap” approaches to create a new large flap. The total area of the jigsaw puzzle flap formed after recombination was slightly larger than the defect, which was conducive to tension-free wound repair. The perforator must be designed in an eccentric manner (located on the side of the flap) to form a perforator propeller flap, V-Y advancement flap, or rotation flap based on the freestyle perforator (Figures 1, 2).

#### Flap Harvest

The key to the harvest of free perforator flap is to find and retrograde dissect of the perforator. The excision was often carried out from the skin to the deep fascia or muscle membrane layer along one side of the designed flap. Meanwhile, the excision position was located between two selected perforators to ensure the perforators could be detected. To dissect perforators easily, silk suturing was used to stitch the whole layer of tissue on one side of the flap to traction the flap and avoid detachment between the superficial layer and the deep layer of the flap. Furthermore, to find the perforator easily, carefully lifting the flap at the deep fascia level and maintaining a certain degree of tension were necessary. More important, when the perforator was located (the diameter of the perforator was more than 0.5 mm or the pulsation was significant), it was released and retrograde-dissected with micro-scissors to retain the width of the fascial pedicle, combined with an appropriate length of the pedicle to transfer the freestyle perforator flap freely.

As this type of perforator flap usually requires a large range of rotation angle, the perivascular tissue (fascial tissue) that may affect the blood supply of the flap after rotation must be completely released to account for the shortcoming that the freestyle perforator may not be separated to sufficient length. For perforator flaps that to filled the deep cavity of the defect, the epidermis was routinely removed to form a dermal-fat flap supplying by freestyle perforator.

#### Creating a Jigsaw Puzzle Flap

All the component perforator flaps were mobilized to the defect, and we observed whether there is the presence of distortion and bending of the vascular pedicle or vascular disturbance at the distal part of the flap. If the blood supply was poor, we quickly checked the pedicle. If the pedicle was twisted and bent, the perforators were further released to obtain a larger range of rotation angle. If the pedicle was normal, warm saline gauze was used to cover the flap and the pedicle while the changing blood supply of the distal flap was observed. After confirming that the blood supply of all the flaps was adequate, all the small flaps were arranged and overlapped reasonably, then sutured to each other to form a large “jigsaw puzzle flap” to repair the defect. Finally, all the donor sites were closed directly with free tension.

#### Postoperative Treatment

After operation, routine intravenous infusion of dexamethasone, intramuscular injection of papaverine, light pressure bandaging, leaving the observation window of the skin flap, observing the skin flap temperature, color and capillary reaction, if the blood supply is not good, give vasodilator, application of VSD and other symptomatic treatment. Stitches were removed 2 weeks after operation.

#### Postoperative Follow-Up

Three to six months after operation, the function and appearance of the skin flap, the function and appearance of the recipient area, and the recurrence of pressure sores were followed up in the outpatient clinic. And the corresponding nursing guidance was given, such as regular use of triangular occipital side rest to avoid long-term hip compression.

## Results

A total of 30 patients were treated. All 65 flaps survived, and only one healed with a delay due to the volume of effusion under the flap, and it healed 1 month later following drainage, dressing changes, and appropriate local pressure treatment. Flaps were transferred in the forms of propeller flap (*n* = 54), V-Y advancement flap (*n* = 7), and rotation flap (*n* = 4) to repair the large defects. The skin flap was rotated 90–180°to repair the wound, there was no obvious distortion and bending of the vascular pedicle and no vascular disturbance at the distal end of the flap. All of the patients achieved satisfactory single-stage reconstruction and all donor sites were closed directly. After follow-up, which ranged from 3–24 months (averaging 6 months), the appearance and function of all flaps were satisfactory for both patient and surgeon. One patient had local decubitus ulcer recurrence 6 months after surgery due to improper care (do not help the patient turn over his body frequently and local pressure prevention). Then, it leads to the recurrence of pressure sores.

### Typical Case

A male patient, 68 years old, had suffered from a sacrococcygeal ulcer for 3 years that was diagnosed as a sacral decubitus ulcer. He was bedridden for 5 years due to a stroke and result in severe craniocerebral limitations, which made the soft tissue of the sacral tail gradually necrotic. As the cystic cavity-type decubitus formed, he was admitted to the hospital and received surgical treatment to repair the decubitus.

Due to the loose subcutaneous tissue. After the necrotic tissue liquefies and falls off, the ulcer with small mouth and large bottom is formed, and the edge is latent. His sacrococcygeal decubitus was flask-shaped, with a soft tissue defect of 9.5 × 10.0 cm and a depth of 3.5 cm. Necrotic sacrum, coccyx, and adjacent tissue were visible in the cystic cavity. Scar tissue with poor blood supply was found on the edge of the ulcer. All the perforators around the defect were detected with portable ultrasound Doppler before surgery. Adjacent zones with the fastest blood flow velocity (more than 2.5 cm/s) and the best soft tissue mobility were selected as the preferred donor area for freestyle perforator flaps. Two freestyle perforator flaps were designed to repair the defect; one of them was removed the epidermis from the distal two-third of the flap and then used to fill the bottom of the defect. We completely removed the necrotic and inactivated tissues around the defect including scars and fibrotic tissues. Until the wound basement is flat and fresh, the size of the defect was about 10.5 × 11 × 3 cm. We constructed the jigsaw puzzle flaps based on the identified freestyle perforators and repaired the defect in a one-stage surgery. We harvested the two freestyle perforator flaps located on the left and right buttocks (one was 7.5 × 12.5 cm and the other, 8.0 × 14.0 cm). The epidermis on the distal two-third of the smaller flap was removed and transferred to the bottom of the defect while bellowing the larger flap. Finally, the perforated flaps were folded together to form a three-dimensional puzzle flap to repair the cavity defect of the decubitus ulcer on the buttocks. Both flaps were transferred in the form of propeller flaps and the donor sites were directly closed. All the flaps survived well after surgery, and patients were satisfied with the function and appearance. There was no ulcer recurrence at a one-year follow-up (Figures 1, 2).

## Discussion

Decubitus ulcers are commonly treated in burn and plastic surgery departments, and the most preferred treatment is reconstruction of the defect by flap or myocutaneous flap. The potential dead space formed by an infection usually exists in cystic cavity-type decubitus ulcers. Due to long-term inflammatory reaction, edema, fibrosis, and surrounding scar tissue with poor blood supply associated with decubitus ulcers, the resulting transplant bed is in a poor condition. The sac wall and scar tissue with poor blood supply must be debrided, which leads to the formation of a deeper defect. Therefore, a large flap with a good blood supply is required to fill and repair the defect. Single or comprehensive use of local skin flaps, myocutaneous flaps, free skin flaps, and other technologies are common surgical approaches. Due to the ratio of length to width, rotation angle, and reliability of blood supply, local random flaps is limited in repairing decubitus ulcers. In addition, when repairing a large defect, it is also difficult to close the donor area of the local random flaps (23). Axial flaps with a reliable blood supply such as myocutaneous flaps and free flaps have achieved good clinical effects. Free flaps such as lateral sacral artery perforator flaps, free perforator propeller flap from buttock and superior gluteal artery perforator “buddy flap” can be used to repair pressure sores (24). However, cystic cavity-type decubitus ulcers, long disease course, copious wound exudation, and serious loss of protein lead to deconditioning in patients who may not easily tolerate the complex reconstruction scheme and trauma of surgery (25–27). Additionally, microsurgery has many problems such as a high technical threshold and high risks. Therefore, designing a construction scheme with a large volume of transplantation tissue, a high safety of blood supply, and less trauma is an urgent need to repair cystic cavity-type decubitus ulcers.

### Freestyle Perforator Flap

The development of research on freestyle perforator flaps is the primary basis for the design of this study. The research progress in freestyle perforator flap use has resulted from the wider clinical applications of perforator flaps and the development of high-sensitivity and portable hand-held Doppler ultrasound devices (18–20). Perforator flaps refer to flaps that supply blood by perforator vessels with a fine diameter, which subvert the traditional concept that muscle and deep fascia are the bases of flap survival. The flap retains the main arteries and muscles maximally in the donor sites, which greatly improves the postoperative appearance and reduces functional injury. Therefore, this method has recently become an important choice for reconstructive surgery (15, 28, 29). With the development of the perforator location technique, it is not necessary to discern traditional anatomical landmarks and anatomical variation limitations. Instead, surgeons design the freestyle perforator flap according to the preoperative detection signal of perforator blood flow around the defect by Doppler ultrasound. Freestyle perforator flaps with different shapes and a large range of mobility can be formed with the perforator as a pedicle on any part of the body. The signal of perforators can be detected by Doppler device or located through imaging. However, freestyle perforator flaps have been traditionally used to transfer and repair small defects. It is difficult to repair a large defect with a single freestyle perforator flap for two major reasons: (1) the size of a single freestyle perforator flap is limited; and (2) if the size of the flap is large, it is difficult to close the donor sites directly, which may then require a free skin graft to repair. The approach described in this paper was applied to repair the large defect resulting from decubitus ulcers on the buttocks. Therefore, we designed two to three local freestyle perforator flaps around the defect, based on the preoperative signal detected by Doppler ultrasound, transferred the flaps to the defect in different styles, and formed a large chimeric flap to cover the defect (30, 31). This method provides a new, safe and easy-to-implement procedure for the reconstruction of large defects resulting from decubitus ulcers.

### Perforasome

The concept of the perforasome and the study of vascular anatomy provide a major foundation for the design of this project. Saint Cyr et al. (32) defined the unique vascular territory of a single perforator as a perforasome. Each perforasome is linked with an adjacent perforasome by linking vessels with tiny diameters (29). The function of linking vessels is highly similar to choke vessels. A choke vessel is the major connection tube between angiosomes that acts as the physiological “demarcation line” of the adjacent angiosome. Numerous animal experiments and clinical experiences have shown the safety area of a flap that is a perforator can supply blood to the perforasome itself and the adjacent one, once a flap has been harvested with one perforator as pedicle along one orientation across three perforasomes (32, 33). The sizes of the flaps designed in this paper were based on the concept of the perforasome. Therefore, the extreme limit of safety for a freestyle perforator flap is the perforasome itself and its adjacent perforasome.

### Local Condition of the Defect

Abundant soft tissue with a satisfactory range of mobility around the defect is one of the necessary anatomic conditions for this procedure. The buttocks have many potential donor sites with a large amount of tissue and a good range of mobility around the defect, which facilitates direct closure of donor sites. Thus, this characteristic local condition creates the possibility of designing multiple flaps freely and forming a puzzle flap to repair a large defect. Importantly, we avoided the superposition of tension resulting from the closure of all donor sites in our design of the jigsaw puzzle flap.

### Application Experience of the Project

The free-style perforator flap technique can be applied in various forms, such as a propeller flap, rotation flap, and V-Y advancement flap, although the selection principles differ for each type of flap. Propeller flaps are recommended for the repair of decubitus ulcers. Surgeons recommend to avoid V-Y advancement flaps as much as possible. This is because decubitus ulcer formation involves a pathophysiological process of ischemic tissue necrosis caused by longstanding local compression, and the marginal fibers of the ulcer and poor blood supply are more significant on histopathologic analysis. If a V-Y advancement flap is used to repair the defect, the edge of the ulcer as the distal part of the advance flap will be directly sutured with the edge of the ulcer in the recipient site, which will delay wound healing and may even contribute to non-healing of the surgical site. Analyzed of the hospitalization time of patients receiving V-Y advancement flaps with propeller and rotation flaps in our clinical practice has shown a longer duration associated with V-Y advancement flaps. The average postoperative stay of propeller and rotation flaps lap is 2 weeks. However, the average postoperative stay of VY-advance flap is 3.5 weeks. There are several possible reasons for this finding. After a propeller flap is transferred, one side of the flap comprises normal tissue with a better blood supply, which provides a good basis for the revascularization of the wound, meanwhile, the transfer of this healthy tissue results in local “biological cleaning” and reduces the probability of infection. Additionally, harvesting the vascular pedicle of a jigsaw puzzle flap without torsion, compression and traction is the primary requirement, according to the position of the rotation point and the length of the dissected vascular pedicle.

During surgery, norepinephrine (1:200,000 units) was injected in the incised tissue to reduce bleeding and maintain a clear field of vision. A pen-tip electrotome was used in a small energy state to harvest the flaps and promote timely hemostasis to facilitate the process of finding and dissociating perforators.

Because soft tissue defects in decubitus ulcers are usually large, there is more effusion in the initial stage after flap transfer and repair. Therefore, after the flap has been transplanted, it is necessary to place a negative pressure drainage at the bottom of the cavity and drain the effusion in a timely manner and locally apply a light pressure bandage to promote the establishment of an adequate blood supply between the flap and the repaired defect.

## Conclusion

The jigsaw puzzle flap, based on a freestyle perforator design concept, makes full use of the anatomical characteristics of the large volume of tissue on the buttocks surrounding the ulcerative defect. Surgeons who apply the theory of freestyle perforator in decubitus ulcer repairing no longer consider the vessel shape and classification of the traditional perforator in the flap, instead, focus on the safety boundary and quantity of flap, which simplifies the flap design process. The jigsaw puzzle flap can repair the large skin and soft tissue defects of cystic cavity-type decubitus ulcers on the buttocks, and the donor flap area can be directly closed. This method is easy to implement, ensuring a safe blood supply and minimal trauma resulting from the avoidance of microvascular anastomosis construction and the conventional myocutaneous flap surgery. The jigsaw puzzle flap is a good alternative approach for decubitus ulcer reconstruction. However, how to reduce the scars between the small perforator flaps that make up the jigsaw puzzle flap needs further study.

## Data Availability Statement

The raw data supporting the conclusions of this article will be made available by the authors, without undue reservation.

## Ethics Statement

The studies involving human participants were reviewed and approved by China National Nuclear Corporation 416 Hospital. The patients/participants provided their written informed consent to participate in this study. The animal study was reviewed and approved by China National Nuclear Corporation 416 Hospital. Written informed consent was obtained from the individual(s) for the publication of any potentially identifiable images or data included in this article.

## Author Contributions

DY participated in the design of the operation and study. XC, BH, and HX carried out the operation and helped to draft the manuscript. LA took charge of collecting the patients' data and helped to perform the operation. WS followed up all the patients, analyzed the data, and collected important background information. All authors read and approved the final manuscript.

## Funding

This work was supported by the National Natural Science Foundation of China (32071238 and 82073477).

## Conflict of Interest

The authors declare that the research was conducted in the absence of any commercial or financial relationships that could be construed as a potential conflict of interest.

## Publisher's Note

All claims expressed in this article are solely those of the authors and do not necessarily represent those of their affiliated organizations, or those of the publisher, the editors and the reviewers. Any product that may be evaluated in this article, or claim that may be made by its manufacturer, is not guaranteed or endorsed by the publisher.
